# Salivary pellicles on titanium and their effect on metabolic activity in *Streptococcus oralis*

**DOI:** 10.1186/1472-6831-13-32

**Published:** 2013-07-16

**Authors:** Marjan Dorkhan, Gunnel Svensäter, Julia R Davies

**Affiliations:** 1 Department of Oral Biology, Faculty of Odontology, Malmö University, Malmö SE-20506, Sweden

**Keywords:** Bacteria, Microbial biofilm, Dental implant, Streptococci

## Abstract

**Background:**

Titanium implants in the oral cavity are covered with a saliva-derived pellicle to which early colonizing microorganisms such as *Streptococcus oralis* can bind. The protein profiles of salivary pellicles on titanium have not been well characterized and the proteins of importance for binding are thus unknown. Biofilm bacteria exhibit different phenotypes from their planktonic counterparts and contact with salivary proteins may be one factor contributing to the induction of changes in physiology. We have characterized salivary pellicles from titanium surfaces and investigated how contact with uncoated and saliva-coated titanium surfaces affects metabolic activity in adherent cells of *S*. *oralis*.

**Methods:**

Salivary pellicles on smooth titanium surfaces were desorbed and these, as well as purified human saliva, were subjected to two-dimensional gel electrophoresis and mass spectroscopy. A parallel plate flow-cell model was used to study binding of a fresh isolate of *S*. *oralis* to uncoated and saliva-coated titanium surfaces. Metabolic activity was assessed using the *Bac*Light CTC Vitality Kit and confocal scanning laser microscopy. Experiments were carried out in triplicate and the results analyzed using Student’s *t*-test or ANOVA.

**Results:**

Secretory IgA, α-amylase and cystatins were identified as dominant proteins in the salivary pellicles. Selective adsorption of proteins was demonstrated by the enrichment of prolactin-inducible protein and absence of zinc-α_2_-glycoprotein relative to saliva. Adherence of *S*. *oralis* to titanium led to an up-regulation of metabolic activity in the population after 2 hours. In the presence of a salivary pellicle, this effect was enhanced and sustained over the following 22 hour period.

**Conclusions:**

We have shown that adherence to smooth titanium surfaces under flow causes an up-regulation of metabolic activity in the early oral colonizer *S*. *oralis*, most likely as part of an adaptation to the biofilm mode of life. The effect was enhanced by a salivary pellicle containing sIgA, α-amylase, cystatins and prolactin-inducible protein which was, for the first time, identified as an abundant component of salivary pellicles on titanium. Further studies are needed to clarify the mechanisms underlying the effect of surface contact on metabolic activity as well as to identify the salivary proteins responsible for enhancing the effect.

## Background

The human oral cavity harbours a large number of different bacterial species which are found in complex, multi-species biofilms. On teeth, these biofilms are commonly known as dental plaque. Critical to the formation and development of plaque is the adherence of pioneer species such as *Streptococcus oralis*, *Streptococcus mitis* and *Streptococcus gordonii* as well as *Actinomyces naeslundii* to the salivary pellicle which coats the tooth surface [1,2]. Once biofilm formation has been initialized and the nascent tooth surface is colonized, co-adherence of later colonizers leads to the formation of mature oral biofilms [3]. The early development of biofilms on dental implants has not been well characterized but the sequence of microbial colonization is thought to be similar to that for teeth in the same oral cavity [4,5].

Teeth and dental implants, as well as the mucosal surfaces, are covered with a pellicle which is a thin film of adsorbed proteins mainly derived from saliva. Pellicle proteins provide an array of potential receptors for the attachment of the early colonizers. A combination of *in vivo* and *in vitro* studies using antibody-based and proteomics approaches, has shown that the acquired enamel pellicle contains a range of different salivary proteins including lysozyme, histatins, statherins [6], α-amylase, cystatins, secretory IgA (sIgA), lactoferrin and proline-rich proteins (Prps) [7] as well as the large salivary mucin, MUC5B [8]. For a comprehensive summary of proteins detected in enamel pellicles see Siquiera *et al*., 2012 [9]. The composition of an adherent pellicle, as well as the density and conformation of the proteins present in it, is generally thought to be influenced by the physico-chemical properties of the substratum but, as yet, the overall composition of the salivary pellicles formed on differently modified titanium surfaces are unknown. Despite the existence of only a few studies, some salivary proteins including cystatins, sIgA, α-amylase and proline-rich proteins have been identified in the adherent pellicle formed on titanium *in vitro* using Western blotting [10,11]. However in all such studies, the methods used to prepare saliva for use as a pellicle can have a large impact on the results obtained. For instance, filtering and centrifugation techniques may remove major populations of salivary proteins leading to the formation of salivary pellicles which are not representative of those present *in vivo*.

The recognition that microbial biofilms are an important factor associated with the failure of dental implants [12] has led to many investigations of bacterial adhesion to titanium surfaces. *In vivo*, where adherence of bacteria and salivary pellicle formation occur in parallel, *S*. *oralis* and *S*. *mitis* were amongst the predominant early colonizers on titanium-coated glass surfaces and no *Actinomyces* species were found [13]. *In vitro*, the presence of saliva on both smooth or moderately-rough surfaces has been shown to both increase and decrease the adherence of the early colonizer, *S*. *oralis*, [10,14] while binding of *A*. *naeslundii* to titanium was unaffected by the presence of a salivary pellicle [11]. Overall, the results of studies of bacterial adherence to titanium in the presence of saliva have not yielded a clear picture and while some of the differences seen may attributable to the saliva used, variation in the bacterial strains and types of titanium surface may also contribute to the lack of consensus.

While biofilm development is important for the development of oral disease, a crucial contributory factor is the physiology and level of activity of the adhered bacteria. Bacterial adaptation to the biofilm mode of life is known to be associated with major changes in transcription and protein synthesis [15]. For example, in *Porphyromonas gingivalis* comparative transcriptomic analysis revealed that a large number of genes are differentially expressed in biofilm cells compared to their free-floating counterparts [16]. In a study in *Streptococcus mutans*, the relative rate of synthesis of at least 25 different proteins was enhanced within 2 hours of attachment to a glass surface. These proteins were mostly associated with carbohydrate catabolism [17] suggesting that changes in metabolic activity may occur during adhesion to surfaces. Little is currently known, however, about the metabolic status of cells during interactions with pellicle proteins in the early stages of biofilm formation. The aim of this work was to study how adherence to titanium surfaces affects the metabolic activity of the early colonizer *S*. *oralis* and to determine the effect of a salivary pellicle on this process. To shed light upon which salivary proteins may influence adherence and metabolic activity, the predominant proteins present in a salivary pellicle formed on titanium have been identified.

## Methods

### Bacteria and culture conditions

A fresh clinical isolate of *S*. *oralis* (89C) was obtained from a patient with an on-going peri-implant infection after ethical approval had been obtained from the Faculty of Odontology [14]. Bacteria were grown overnight on blood agar in an atmosphere of 5% CO_2_ in air at 37°C. Colonies were suspended in 120 ml phosphate buffered saline [0.15M NaCl, 10mM NaH_2_PO_4_, pH 7.4 (PBS)] to give an OD_600nm_ = 0.6. For the flow-cell experiments, an equal volume of PBS was added to halve the cell concentration prior to biofilm formation, whereas for the planktonic experiments the original bacterial suspension was mixed with an equal volume of either PBS, or 50% whole human saliva to give a final concentration of 25% saliva.

### Collection and preparation of saliva

Whole saliva collected on ice over 1 hour from ten healthy individuals was pooled and prepared as described previously [18] after ethical approval had been obtained from the Faculty of Odontology. Briefly, the sample was mixed with an equal volume of PBS, stirred gently overnight at 4°C and centrifuged in a Beckman Coulter Avanti J-E centrifuge (Beckman JA 20 rotor; Beckman Coulter, Brea, CA) (20 minutes, 30 000 *g*, 4°C). The supernatant was then subjected to isopycnic density-gradient centrifugation in CsCl/0.1M NaCl in a Beckman Coulter Optima LE-80K Ultracentrifuge (Beckman 50.2 Ti rotor, starting density 1.45 g ml^−1^) at 36000 rpm for 90 hours at 15°C. Fractions containing bacteria were discarded and those remaining were pooled, dialysed against PBS and stored at − 20°C.

### Titanium surfaces

The titanium surfaces used in this study were of commercially pure grade IV titanium, which was smooth, with an average surface roughness (S_a_) of 0.1 μm [14]. The plates (99 × 25 × 0.8 mm) were turned, cleaned with detergent, rinsed with distilled water and sterilized using γ irradiation (ELOS Pinol A/S).

### Characterization of saliva pellicles

Two titanium plates, separated by a rubber spacer with thickness of 1.6 mm, were mounted in a flow-cell and the surfaces coated with 50% whole human saliva overnight. After this time the flow-cells were drained and the surfaces washed (2 × 2 mins) with PBS on a rocking plate. To remove the surface-associated pellicles, a mixture of Tween 80 (0.006 v/v%) and Triton X-100 (0.012 v/v%) was introduced and the whole flow-cell placed in an ultrasonic bath for 1 hour. The contents were then drained and collected before repeating this step for an additional 15 minutes. Protein desorbates collected after each wash were pooled and the protein concentration determined using a 2D Quant kit (GE Healthcare Life Sciences). A volume corresponding to 20 μg protein was subjected to 2DE. Briefly, the desorbate was diluted with rehydration buffer and placed in a re-swelling cassette with 18 cm pH 4–7 linear IPG strips (GE Healthcare Life Sciences) on top. Rehydration was undertaken at room temperature for 30 hours under silicone oil. Isoelectric focusing was carried out using a Multiphor II (GE Healthcare Life Sciences) with cooling water at 15°C supplied by Pharmacia Multitemp II. The focusing was initiated at 150 V for 1 hour and continued at 300 V for 3 hours, 600 V for 3 hours, 1200 V for 12 hours and finally 3,500 V for 20 hours. After focusing, the IPG strips were stored at −80°C. Before running in the second dimension, the IPG strips were equilibrated first in 50 mM Tris buffer pH 6.8 containing 2% SDS, 26% glycerol and 16 mM DTT for 15 minutes and then in 50 mM Tris buffer pH 6.8 containing 2% SDS, 26% glycerol, 250 mM iodoacetamide and 0.005% bromophenol blue for another 15 minutes. The equilibrated IPG strips were embedded on top of 14% polyacrylamide gels (20 × 20 × 0.1 cm) using 0.5% (w/v) molten agarose. SDS-PAGE was performed at a constant current of 15 mA gel^-1^, 10°C, overnight in a PROTEAN II xi cell (Bio-Rad) with rainbow high-range molecular mass standards (GE Healthcare Life Sciences) run on the acidic side of the IPG strips. Gels were stained with Coomassie brilliant blue or silver according to the protocols from GE Healthcare Life Sciences.

### Identification of proteins on 2D gels by LC-MS/MS

Spots of interest were excised manually from Coomassie brilliant blue stained 2DE gels of whole saliva and subjected to LC-MS/MS as described previously [19]. Briefly, proteins were reduced with DTT (60°C, 20 minutes), alkylated with iodoacetamide (25°C, 10 minutes) and then digested with trypsin (37°C, 8 hours). Tryptic peptides were separated and subjected to MS. Peptide peaks were deconvoluted automatically and mass lists in the form of Mascot Generic Files used as the input for Mascot MS/MS Ions searches of the NCBInr database using the Matrix Science web server (http://www.matrixscience.com).

### Determination of surface coverage and viability

Viability of cells suspended in PBS or 25% whole saliva was assessed by staining a drop of the suspension with the Live/Dead *Bac*Light staining kit (Life Technologies, Stockholm, Sweden) at baseline (time 0), after 2 and 24 hours and viewing with an inverted confocal laser scanning microscope (CSLM) (Eclipse TE2000, Nikon Corp.). The vertical, parallel plate flow-cell system used has been described previously (14). Briefly, *S*. *oralis* cells were passed over two titanium surfaces (99.25 × 25.25 × 0.8 mm) separated by a 1.6 mm rubber spacer, which were either uncoated or had been coated with saliva overnight. All experiments were carried out at 37°C and a laminar flow of 42 ml h^-1^ was used to model the daily flow of saliva over the oral surfaces. All solutions were introduced through the lower inlet and outflow occurred through the upper valve. Initially, surfaces were rinsed with PBS for 30 minutes. The same bacterial suspension was then introduced into two flow-cells; one containing two uncoated surfaces and the other containing two saliva-coated surfaces for 2 or 24 hours at 37°C. After this time, the flow-cells were washed with PBS (as above) for 30 minutes to remove loosely attached bacteria from the surfaces. Surface coverage and viability of surface-associated cells were assessed on one of the titanium plates in each flow-cell using Live/Dead *Bac*Light staining. Experiments were carried out three times using independent bacterial cultures.

### Determination of metabolic activity

To investigate metabolic activity of planktonic cells, an aliquot was removed from the same bacterial suspension used for the viability measurements, placed in an ibidi flow-cell chamber and the cells incubated with the *Bac*Light CTC Vitality Kit (Life Technologies, Stockholm, Sweden) in a humid chamber at 37°C for 2 hours. The slides were then viewed using a CSLM. To investigate the metabolic activity of adhered cells, the second titanium plate in each flow-cell, was incubated with the *Bac*Light CTC Vitality Kit as above and the cells then counterstained with 4′, 6-diamidino-2-phenylindole (DAPI, Life Technologies, Stockholm, Sweden). Stained cells were visualized using a CSLM.

### Image analysis and statistics

For samples stained with the Live/Dead *Bac*Light staining kit, ten random images each with an area of 127.3 μm^2^ were taken for image analysis. Images were analysed using the *bioImage*_*L* software package to quantitate the average surface coverage as well as the proportion of live (green) and dead (red) cells [20]. For samples stained with the *Bac*Light CTC Vitality Kit to assess metabolic activity, image analysis was performed by observing at least 1000 cells and counting the number of metabolically active cells (red/pink) and non-active cells (unstained in the suspension samples or counterstained blue on the titanium surfaces). The results obtained were evaluated using Student’s *t*-test to compare two groups or a one-way ANOVA with the Bonferroni post-test to compare three groups. A confidence interval of 95% was chosen and p values below 0.05 were considered significant.

## Results

### Biofilm formation on titanium in flow-cells

**Figure 1:**
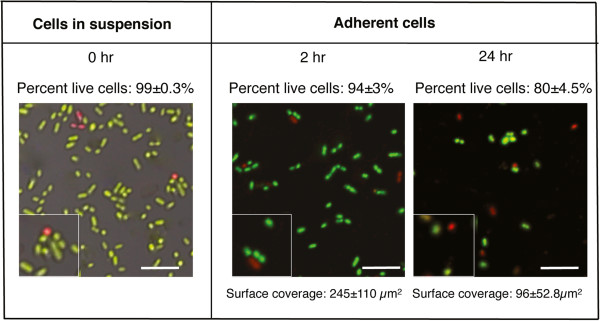
**Images showing biofilm formation by *S. oralis *on uncoated titanium surfaces.** Bacteria suspended in PBS were allowed to form biofilms on titanium surfaces in a parallel plate flow-cell system for 2 and 24 hours. The viability of the cells was assessed by Live/Dead *Bac*Light staining and viewing with CSLM. Surface coverage on the titanium plates was assessed from ten random images using the *bioImage*_*L* software package. The scale bars represent 10 μm and the inserts show cells in twofold enlargement.

The biofilm-forming ability of *S*. *oralis* was investigated in a flow-cell system containing two uncoated titanium surfaces. Bacterial colonies dispersed in PBS were introduced into flow-cells and allowed to adhere for 2 or 24 hours. After 24 hours, the average surface coverage was decreased by 60% compared to that after 2 hours, suggesting that some of the cells which adhered initially detached over time (Figure 1). In the initial cell suspension the level of viability was high as revealed by staining with the *Bac*Light Live/Dead kit. After 2 and 24 hours, the viability of the adherent populations was not significantly different to that of the original suspension, indicating that binding to titanium did not adversely affect the cells. Since surfaces in the oral cavity are covered with a salivary pellicle, we investigated the effect of saliva on adherence and viability of *S*. *oralis*. On surfaces coated with 25% saliva, the average surface coverage after 2 hours (178 ± 103 μm^2^) was not significantly different to that seen on the uncoated surfaces (p = 0.67) (data not shown). As for the uncoated surfaces, after 24 hours the average surface coverage on the saliva coat had declined (24.6 ± 7.4 μm^2^), demonstrating that bacterial cells also detached from these surfaces over time. The level of viability of the cells on the saliva-coated surface was not significantly different to that on the uncoated surface at the same time point.

### Metabolic activity in relation to contact with uncoated and saliva-coated surfaces

**Figure 2:**
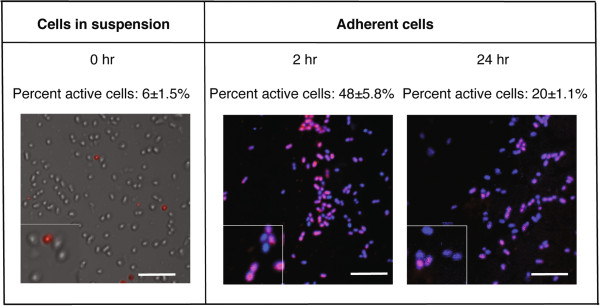
**Images showing metabolic activity of *S. oralis *in suspension and adhered to uncoated titanium surfaces.** Bacteria suspended in PBS were allowed to form biofilms on titanium surfaces in a parallel plate flow-cell system for 2 and 24 hours. The metabolic activity of the cells was assessed using the *Bac*Light CTC Vitality Kit. For adhered cells, DAPI was used as a counterstain. Cells were viewed with CSLM and the proportion of metabolically active (red/pink) cells assessed by manually counting at least 1000 cells. The scale bars represent 10 μm and the inserts show cells in twofold enlargement.

Metabolic activity of *S*. *oralis* cells was assessed using the *Bac*Light CTC Vitality Kit, where metabolically active cells reduce the colourless tetrazolium salt to an insoluble formazan product causing them to appear red (or pink in the presence of the blue DAPI counterstain). Cells removed from blood agar at time 0 and dispersed in PBS showed a low level of endogenous metabolic activity (6 ± 1.5%) (Figure 2). Continued incubation of the cells in PBS had no significant effect on the level of metabolic activity after 2 hours (4 ± 0.5%) or 24 hours (2 ± 0.1%). However, after 2 hours in the flow-cell model, the adherent population contained a significantly higher proportion of red cells indicating that metabolic activity was stimulated by contact with a surface. After 24 hours, the level of metabolic activity within the adherent population had decreased but was still significantly greater than in the original PBS suspension (p < 0.01).

**Figure 3:**
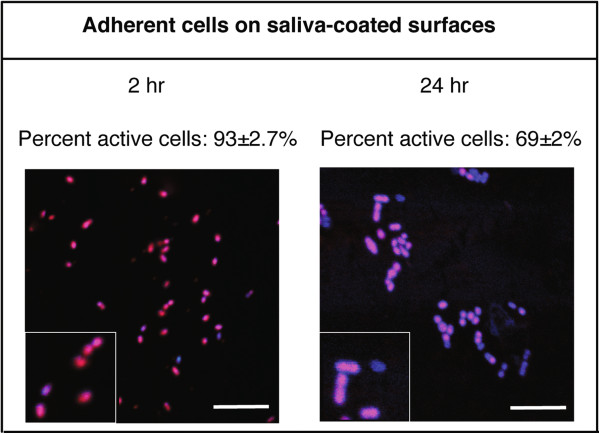
**Images showing metabolic activity of *S. oralis *adhered to saliva**-**coated titanium surfaces.** Bacteria suspended in PBS were allowed to form biofilms on saliva-coated titanium surfaces in a parallel plate flow-cell system for 2 and 24 hours. The metabolic activity of the cells was assessed using the *Bac*Light CTC Vitality Kit with DAPI as a counterstain. Cells were viewed with CSLM and the proportion of metabolically active (red/pink) cells assessed by manually counting at least 1000 cells. The scale bars represent 10 μm and the inserts show cells in twofold enlargement.

The effects of saliva coating on the metabolic activity of adherent bacteria were then investigated. This revealed that the levels were significantly higher for cells associated with the saliva-coated surface after 2 hours and 24 hours compared to those in the original PBS suspension (p < 0.001) (Figure 3). Incubation of bacteria with 25% saliva in suspension caused no significant change in metabolic activity over 2 hours (4 ± 0.5%) or 24 hours (3 ± 0.6%) suggesting that the effect was specific to salivary proteins adhered to a surface. These data thus show that adsorbed salivary proteins have the capacity to elicit a metabolic response that is not seen when the proteins are present in solution.

Since non-viable cells on the surfaces are not expected to show metabolic activity, to investigate the level of metabolic activity as a function of the number of viable cells, a ratio was calculated for each time point (Table 1). This revealed that for cells suspended in PBS or saliva, while viability was maintained, there were no significant changes in metabolic activity over time. Adherence to an uncoated surface caused the proportion of the viable cells that were metabolically active to rise to 50% after 2 hours, whereas binding to a saliva pellicle significantly increased this level to 98% of the viable cells (p < 0.001). Thus while initial contact with a surface caused an increase in metabolic activity within the population, this was greatly enhanced by the presence of a salivary pellicle. After 24 hours, the proportion of metabolically active cells on the uncoated titanium surface had decreased to 25% whereas on the saliva-coated surface the level remained at 96%. This suggests that, in addition to enhancing the initial response to surface contact, the presence of salivary proteins sustained the increase in metabolic activity over 24 hours.

**Table 1:** Proportion of viable bacteria in the population showing metabolic activity under different conditions

**Environment**	**% live cells with metabolic activity**		
	**0 h**	**2 h**	**24 h**
Cells suspended in PBS	6 ± 1.5	4 ± 0.5	2 ± 0.03
Cells adhered to uncoated surfaces in PBS	-	50 ± 4.5	25 ± 2.6
Cells suspended in 25% saliva	-	4 ± 0.8	3 ± 0.6
Cells adhered to surfaces coated with 25% saliva	-	98 ± 4.3	96 ± 6.3

### Characterisation of saliva coating on titanium surfaces

**Figure 4:**
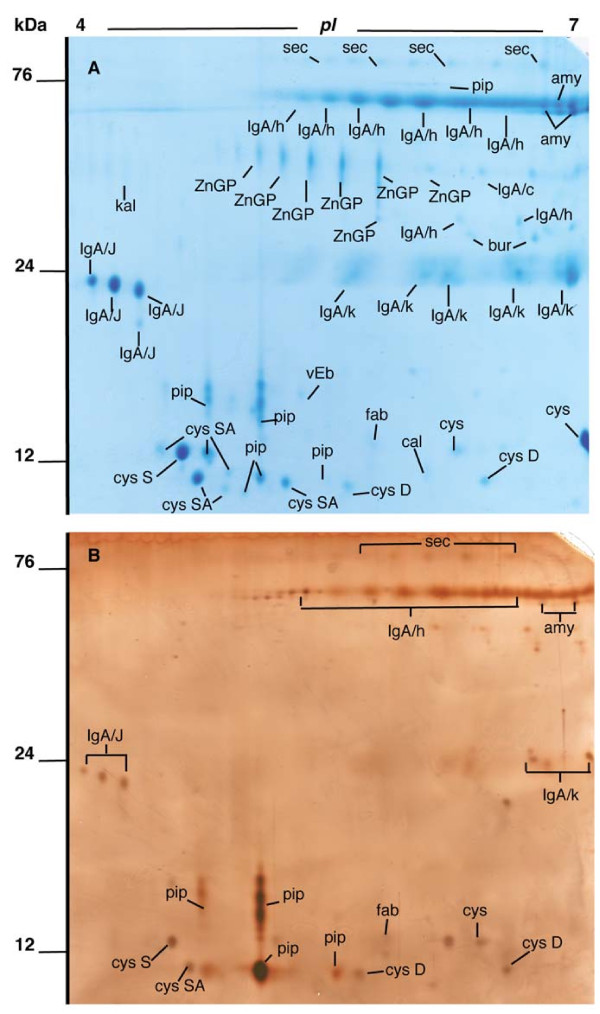
**2DE gels of whole saliva and the salivary pellicle desorbed from titanium surfaces.** Pooled whole human saliva **(A)** and salivary pellicles desorbed from titanium surfaces using detergent **(B)** were subjected to isoelectric focussing on pH 4–7 IPG strips followed by SDS-PAGE on 14% gels. Gels were stained with Coomassie Blue **(A)** or silver **(B)**. Spots of interest were picked from the Coomassie gel, identified using LC-MS/MS and spot identities transferred to the silver gel. An explanation of the labels is given in Table 2.

To identify the major protein components in the bacteria-free saliva preparation, the material was subjected to 2DE and proteins visualised by staining with Coomassie brilliant blue (Figure 4a). This revealed the presence of over 100 spots, of which the majority (70) were picked, and 68 of these could be identified using LC-MS/MS (Table 2). Almost all the proteins present were shown to be of salivary origin, with secretory IgA, zinc-α_2_-glycoprotein, members of the cystatin family, α-amylase and prolactin-induced protein (PIP) as the dominant species in the preparation. In addition, kallikrein, fatty acid-binding protein and von Ebner’s protein were identified.

**Table 2:** Identities of proteins from whole saliva or salivary pellicles desorbed from titanium surfaces using detergent obtained using LC-MS/MS of spots from 2DE gels.

**Protein identity**	**Spot name**	**% Sequence coverage**	**Detected on surface**
**Amylase**	amy	45-55	Yes
**Calgranulin**	cal	49	No
**Cystatin**			
S	cysS	77	Yes
SA	cysSA	37–73	Yes
D	cysD	31	Yes
**Fatty acid binding protein**	fab	51	Yes
**Immunoglobulin A**			
J chain	IgA/J	37–63	Yes
Heavy chain	IgA/h	18–36	Yes
Heavy chain C region	IgA/c	21	No
Secretory component	sec	32–36	Yes
Bur	Bur	11–15	No
**Immunoglobulin**			
Light chain (kappa)	Ig/κ	38–59	Yes
**Kallikrein**	kal	10	No
**Prolactin**-**inducible protein**	pip	63–67	Yes
**Von Ebners’s gland protein (lipocalin)**	vEb	27	No
**Zinc-α2**-**glycoprotein**	ZnGP	23-45	No

To identify the proteins present in the salivary pellicle formed on titanium, surfaces were incubated overnight with saliva and, after washing, the adhered proteins were desorbed with detergent and subjected to 2DE (Figure 4b). Silver staining of the gels revealed around 50 spots of which all were seen in the Coomassie stained saliva gel. Secretory IgA, cystatin proteins, α-amylase and PIP were present but zinc-α_2_-glycoprotein was absent indicating that this protein did not adhere to the titanium surface. The relative intensity of PIP spots in the pellicle was greater than in the original saliva preparation suggesting that this protein might be enriched on the titanium surface.

## Discussion

Early colonizers such as *S*. *oralis* initiate biofilm formation by interacting directly with the salivary pellicle that is present on oral surfaces. In this study, we have used pellicles of saliva prepared by density-gradient centrifugation under non-denaturing conditions. The advantage of this technique is that salivary bacteria, which are pelleted, can be separated from large macromolecules allowing the preparation of bacteria-free, ‘native’ saliva in which even large salivary proteins are present. We have previously identified the two large salivary mucins (MUC5B and MUC7) as well as gp340, lysozyme, lactoferrin, α-amylase, secretory IgA and statherin in this preparation using ELISA [21]. In this study, we performed 2DE in combination with LC-MS/MS to identify lower molecular-weight salivary proteins (Figure 4a). Secretory IgA was the most abundant protein as revealed by the presence of several fragments (secretory component, heavy chain, κ-chain and J-chain). In agreement with analyses of human saliva by other groups using proteomics approaches [7,22] we were also able to identify α-amylase, proteins of the cystatin family, zinc-α_2_-glycoprotein and PIP. Fatty acid binding protein, kallikrein and von Ebners gland protein (lipocalin) were present in minor amounts. In this study we have applied the methodology to examine the salivary pellicles formed on titanium. This showed that sIgA and α-amylase were the most abundant proteins in the pellicle in addition to members of the cystatin protein family and PIP (Figure 4b). Previous studies using SDS-PAGE combined with Western blot analysis with specific antibodies against salivary proteins, have shown that α-amylase, sIgA and Prps bind to titanium [10,11]. Zinc-α_2_-glycoprotein was absent from the pellicle desorbate suggesting that this protein does not adhere to titanium whereas the greater relative abundance of PIP in the desorbate than in the original saliva preparation indicates that the protein is enriched on the surface. Oral bacteria such as *S*. *salivarius*, *S*. *parasanguinis* and *S*. *oralis* can interact with PIP [23,24], suggesting that this protein could play an important role in modulating bacterial colonization of oral surfaces. To our knowledge, this is the first time that PIP has been identified as an abundant protein in pellicles on titanium. A 20 kDa protein corresponding to PIP [25] has previously been demonstrated to bind to hydroxyapatite but was not enriched in the same way found here [26]. One limitation of this study however is that it is currently unknown whether the results are applicable to other titanium surfaces with differing surface topographies or surface modifications.

In the flow-cell model, *S*. *oralis* adhered well to saliva-coated surfaces after 2 hours - in keeping with other studies on primary colonizers such as *Streptococcus anginosus*, *Streptococcus gordonii* and *Streptococcus sanguinis*[10] and *Actinomyces naeslundii*[11]. As a group, oral streptococci are known to express adhesins which have affinity for a range of proteins present in saliva [27]. In a previous study, we identified a 1060 amino-acid-containing, LPXTG-linked protein expressed in strains of *S*. *oralis* which bound well to salivary pellicles and *in silico* analysis of the *S*. *oralis* genome revealed a further two LPXTG-linked putative adhesins [14]. Little is however known about specific adhesins present on *S*. *oralis* and the ligands to which the previously identified adhesins bind are currently unidentified.

In this study, the fluorescent redox indicator CTC, which gives rise to red, insoluble product when reduced by intracellular electron transport activity, was used as a marker of metabolic activity [28]. This technique has been used previously to investigate the activity of *Staphylococcus aureus* and *Staphylococcus epidermidis* on albumin-coated titanium surfaces [29]. In oral streptococci, the major energy-generating pathway which results in high NADH/NAD + ratios, and thus red staining within the cells, is the glycolytic pathway. We have shown that adherence of cells to uncoated titanium under flow led to an increase in metabolic activity within the viable bacteria population from 6% to 50% within the first 2 hours (Figure 2). This suggests that, even in the absence of nutrients, surface contact activated energy-generating pathways within the cells. During transition from the planktonic to the biofilm mode of life, microorganisms are well known to undergo major transcriptional and proteomic changes [15]. For example, synthesis of a range of enzymes in the glycolytic pathway including dehydrogenases and kinases has been shown to be enhanced during early biofilm formation [17]. In addition, transcriptional studies in *P*. *gingivalis* have shown that 18% of the genome is differentially regulated in adherent cells compared to those in suspension, with changes in expression of genes associated with cell envelope synthesis, DNA replication and metabolism [16]. Since in *E coli*, it has been proposed that the activity of the glycolytic pathway is regulated by the demand for ATP [30], the requirement for energy to drive anabolic processes associated with surface contact could explain the increase in metabolic activity seen in our study. The stimulatory effect of surface contact was doubled by the presence of a saliva coat, where 93% of the viable population was metabolically active after 2 hours (Figure 4, Table 2) and the effect was sustained over the following 22 hours. This increase was not seen in bacteria in contact with the same preparation of saliva in solution, suggesting that the conformation of the proteins is important for the response. Thus we have shown that surface-associated salivary proteins have the capacity to influence the metabolic status of adherent *S. oralis* cells. However, this study is limited by the use of one strain of *S*. *oralis* and further studies are therefore required to determine whether the results can be generalized to other oral bacteria as well as to identify mechanisms underlying the effect and the salivary proteins responsible.

## Conclusions

In conclusion, we have shown that adherence to smooth titanium surfaces is associated with an up-regulation of metabolic activity in the early oral colonizer *S*. *oralis*, most likely as part of an adaption to the biofilm mode of life. The effect was enhanced by the presence of a salivary pellicle which was shown to contain a number of proteins including sIgA, α-amylase, cystatins and PIP which, for the first time, was identified as an abundant component of salivary pellicles on titanium. Further studies are now required to clarify the mechanisms underlying the effect of surface contact on metabolic activity as well as to identify the salivary proteins responsible for the effect.

## Abbreviations

CSLM: Confocal scanning laser microscopy; 2DE: Two-dimensional SDS-Polyacrylamide gel electrophoresis; DAPI: 4′,6-diamidino-2-phenylindole; PBS: 0.15 M sodium chloride, 10 mM sodium dihydrogen phosphate, pH 7.4.

## Competing interests

The authors declare that they have no competing interests.

## Authors’ contributions

MD participated in planning and designing the study, performed most of the laboratory work and participated in the data analysis as well as drafting of the manuscript. GS and JRD participated in study design, data analysis and drafting of the manuscript. All authors have read and approved the final manuscript.

## Pre-publication history

The pre-publication history for this paper can be accessed here:

http://www.biomedcentral.com/1472-6831/13/32/prepub
